# “I Was Thinking About Food All the Time, I Didn’t Have Enough”: Understanding the Multidimensional Nature of Food Insecurity Among Undergraduates at an Urban U.S. Campus

**DOI:** 10.3390/nu18030375

**Published:** 2026-01-23

**Authors:** Gabby Headrick, Julia Blouin, Mackenzie Konyar, Lily Amorosino, Matea Mandic, Anna Razvi, Kaleigh Steigman, Sean Watley, Douglas Frazier, Jennifer Sacheck

**Affiliations:** 1 Milken Institute School of Public Health, George Washington University, Washington, DC 20037, USA; juliab1@gwu.edu (J.B.); lilyamorosino@gwu.edu (L.A.); annarazvi@gwu.edu (A.R.); 2 School of Nursing, George Washington University, Washington, DC 20037, USA; mkonyar654@gwu.edu; 3 Columbian College of Arts & Sciences, George Washington University, Washington, DC 20052, USA; mateamandic01@gwu.edu; 4 Division for Student Affairs, George Washington University, Washington, DC 20052, USA; kaleigh.steigman@gwu.edu (K.S.); sean.watley@gwu.edu (S.W.); douglasfrazier@gwu.edu (D.F.); 5 School of Public Health, Brown University, Providence, RI 02903, USA

**Keywords:** college students, undergraduates, food security, meal plans, college health

## Abstract

**Background:** Food insecurity among college students is a multidimensional challenge shaped by individual, interpersonal, institutional, community, and policy factors. Although many campuses require or provide meal plans, students may experience food insecurity when barriers related to agency (choice and autonomy), utilization (nutrition security), and availability persist. This study explored how undergraduate students at a private, urban U.S. university experience and navigate the multiple dimensions of food insecurity. **Methods:** We conducted in-depth, semi-structured interviews via Zoom between December 2024 and January 2025 with n = 22 undergraduate students recruited based on food security status, determined by a Fall 2024 longitudinal survey using the USDA Six-Item Short Form. Transcripts were double-coded by trained research assistants in ATLAS.ti using an inductive codebook. Thematic analyses followed a phronetic, iterative approach, organizing findings within a socio-ecological determinants framework and comparing themes by food security status. **Results:** We identified nine themes across four domains (individual, interpersonal, institutional and community, and political). At the individual level, constrained personal resources for groceries and cooking, time scarcity leading to skipped meals, and health impacts that detracted from academics emerged as key themes. Interpersonally, reliable family financial support was protective and informal support from peers/coaches filled gaps sporadically for some. At the institutional and community level, dining hall hours misaligned with student schedules, perceived limited variety and nutrition quality reduced food agency and utilization, and transportation impeded use of the sole grocery partner accepting university meal plan benefits. Notably, meal plans including unlimited meal swipes provided stable access but did not guarantee food security when food agency and utilization barriers persisted. Many students relied on campus events for free food; formal assistance (e.g., food pantry) was largely underused. At the policy level, Supplemental Nutrition Assistance Program (SNAP) awareness and enrollment was limited among our sample. **Conclusions:** Meal plan access alone is insufficient to ensure food security. Campus strategies should extend beyond access to prioritize flexibility, variety, and alignment with students’ schedules and preferences, while strengthening communication and eligibility support for external benefits. Future work should design and evaluate interventions that integrate all dimensions of food security and address institutional policies affecting students’ basic needs.

## 1. Introduction

Food insecurity is a significant public health challenge, affecting an estimated 47 million people (13.5% of households) in the United States (U.S.) in 2023 [1]. The U.S. Department of Agriculture (USDA) defines food insecurity as the inability to obtain an adequate, safe, and culturally appropriate diet through socially acceptable means [1]. Food security includes six interconnected dimensions [2,3]: (1) access, which refers to whether individuals can afford food; (2) availability, which considers whether nutritious food is physically reachable; (3) stability, which reflects consistent access to food despite seasonal or economic fluctuations; (4) utilization, which involves achieving nutrition security so that food supports health and prevents disease; (5) agency, which relates to the ability to make autonomous food choices; and (6) sustainability, which addresses whether food systems can meet current and future needs of generations. Vulnerability across these dimensions varies across populations, with college students being particularly at risk due to unique financial and time burdens experienced during their academic careers [4].

Studies estimate that 30–50% of college students across the globe report experiencing food insecurity at some point during this life stage [5,6]. In the U.S., certain college students are at an increased risk for food insecurity including those identifying as Black, Hispanic, or Latino, first-generation college students, financially independent college students (i.e., no financial support from family), and international students [5,7,8,9,10]. For example, an analysis of U.S. nationally representative data from the Panel Study of Income Dynamics between 2015 and 2019 found that 21% of Black students and 26% of Hispanic students experienced food insecurity compared to 9% of white students. Furthermore, 18% of first-generation students experienced food insecurity compared to 10% of non-first-generation students [8]. These disparities mirror those experienced by racialized and economically disadvantaged populations across the U.S. [1,11] However, they warrant special attention as college students represent a growing demographic in the U.S. that faces unique challenges and heightened vulnerability to food insecurity [9,12]. When food insecurity is unaddressed, students report poorer physical health, diet quality, mental health, and academic performance compared to peers experiencing food security [13,14,15].

Determinants of food insecurity among college students operate across multiple levels and intersect with the six dimensions of food security in complex ways [4]. At the individual level, limited financial resources, competing academic and work demands, and inadequate nutrition knowledge or cooking abilities may constrain students’ ability to access and utilize health-promoting foods [16,17]. Interpersonal factors, such as limited family financial support or weak social networks, further exacerbate vulnerability by reducing access to financial assistance and emotional support [4,17,18]. Students without these support systems often face greater challenges in coping with unexpected expenses and maintaining food security [4].

Institutional factors strongly influence college students’ food security across multiple dimensions as college/university campuses are often their primary environment, especially among undergraduates. High costs of tuition, meal plans, and housing affect access, availability, and stability of food throughout the semester [4,9]. Some evidence suggests food insecurity may occur episodically rather than persistently, possibly due to fluctuating institutional resources, though more research is needed [19,20]. While some universities offer food pantries, lack of awareness and stigma often limit use, reducing students’ ability to meet health and food needs [21,22]. Dining systems can also pose barriers, including restricted hours and unappealing options, even for students with meal plans [23,24,25]. These challenges vary widely across institutions, underscoring the need for research on effective strategies to strengthen institutional support [12].

Beyond campus, community-level determinants such as local food environments and transportation infrastructure influence the physical availability of food [26,27]. Higher costs of living in urban areas compared to rural communities may further constrain economic access, although urban settings may offer greater opportunities to access food outside campus [14,28]. Additional research is needed to understand how urbanicity shapes food security among college students [29,30]. At the policy level, restrictive eligibility criteria for federal nutrition assistance programs, particularly the Supplemental Nutrition Assistance Program (SNAP), hinder students’ ability to enroll and benefit from this federal supplemental financial support that can be used to purchase groceries [4]. Poor communication and outreach regarding SNAP eligibility further limit participation among those who qualify [22,31]. Finally, rising tuition and housing costs reduce students’ disposable income for food, forcing trade-offs that increase vulnerability to food insecurity. However, little is known about how students weigh these trade-offs when prioritizing educational expenses over basic needs [9].

Although several qualitative studies have advanced the understanding of the experience of food insecurity among college students, no studies to our knowledge have operationalized the decision-making processes, trade-offs, and lived experiences of college students across the multiple dimensions of food security. Further, resources and practices at private universities to address food insecurity may differ compared to public universities and community colleges, which comprise much of the established evidence about the experience of college food insecurity. To address this gap, the present study draws on in-depth interviews with undergraduate students at a private university in an urban U.S. setting to examine how individual, intrapersonal, institutional, community, and political determinants intersect to influence the multiple dimensions of food security, and how these experiences vary by food security status.

## 2. Materials and Methods

### 2.1. Study Setting

The George Washington University (GW) is a private university located in Washington, D.C., an urban center in the U.S. where the livable annual salary for a household of one with no children is estimated to be $54,034 USD [32]. In 2024, 11,182 undergraduates and 10,839 graduated students attended GW [33]. Full-time undergraduate tuition costs $67,420 per academic year for those enrolling in 2024, with housing and dining costing an additional $16,920 per academic year [34,35]. Among the undergraduate student population, 7% identify as international students, 48% identify as white, 65% received financial aid through grants (i.e., Pell Grants provided to undergraduates with exceptional unmet financial need), and 32% received financial aid through student loans [36].

### 2.2. Sample and Recruitment

In Fall 2024, we invited undergraduate and graduate students attending the university to complete a longitudinal survey assessing self-reported food security status, physical health, mental health, and academic success. Undergraduate and graduate students were eligible to participate if they were 18 years of age or older, attended their degree program part-time or full-time, and completed most (>50%) of their course work in-person during the Fall 2024 semester. We recruited a convenience sample through emails, tabling, and social media outreach.

Students completed the survey at three timepoints (or “Waves”) throughout Fall 2024 to identify how food security status changed over the academic semester. Students first completed Wave 1 of the survey in September 2024, then Wave 2 in November 2024, and Wave 3 in December 2024. In total, n = 422 students completed Wave 1, making them eligible to be invited to complete Wave 2 and Wave 3 (a student could complete Wave 3 without completing Wave 2). In Wave 2, n = 260 students completed the survey (62% retention from Wave 1), and n = 206 students completed Wave 3 (49% retention from Wave 1; 80% retention from Wave 2). Findings from the survey are reported elsewhere [37].

In Wave 3, at the conclusion of the survey, we asked students if they consented to future contact for additional study activities on food resource management and coping strategies; n = 97 students (n = 57 undergraduates and n = 40 graduates) consented. In December 2024, we recruited undergraduates from this consented sample to participate in an in-depth interview; we specifically focused on undergraduates given the differences in lived realities (e.g., enrollment in meal plans, on-campus housing, financial aid, and family support) compared to graduate students. We purposively recruited undergraduates based on their food security status in Fall 2024. Specifically, we recruited those who: (1) experienced food security throughout the entire semester (persistently food secure); (2) experienced food insecurity during at least one time point in the semester but not all three time points (episodically food secure); and (3) experienced food insecurity at all three time points in the semester (persistently food insecure). We invited all n = 57 undergraduates to participate in an interview and contacted them by email up to four times. Due to challenges recruiting students who experienced episodic food insecurity, we collapsed students persistently experiencing food insecurity (n = 9) and students episodically experiencing food insecurity (n = 3) into a single category representing any food insecurity in Fall 2024. While this approach limited our ability to examine stability as a dimension of food security, it allowed for robust analysis across the other dimensions of food security. We conducted interviews until data saturation in the two groups (persistent food security and any food insecurity) was achieved [38].

### 2.3. In-Depth Interviews

Students completed in-depth interviews over Zoom between December 2024 and January 2025 with a trained undergraduate (n = 2) or graduate (n = 1) student research assistant. Research assistants completed a three-hour training with the principal investigator prior to conducting any interviews; this included completing a mock interview with another student (not included in our sample) and receiving feedback on their data collection process from the principal investigator.

Research assistants used a semi-structured interview guide to conduct all interviews. The principal investigator co-developed the semi-structured interview guide in collaboration with the study team, which included the basic needs program associate, the first-generation program associate, and the director of dining services at the university, student volunteers at the university food pantry, and college students with lived experiences of food insecurity. The interview guide consisted of open-ended questions and follow-up probes across three sections focused on: (1) food acquisition habits; (2) food management strategies; and (3) perceived relationships between food access, health, academics and social life (Table 1). Questions were pilot tested for feedback among n = 3 undergraduate students not involved in the study before starting data collection.

Prior to the Zoom interview, the participant provided informed verbal consent to participating in the interview and being audio-recorded. Interviews lasted approximately 40 to 45 min. Following the interview, the participant received a $50 Amazon gift card and a resource flyer describing basic needs support on campus via email. Throughout data collection, the principial investigator met at least once per week with the research assistants to discuss the data collection process and iteratively refined interview guide to best capture emerging themes. This study was approved by the Institutional Review Board (IRB) of George Washington University (IRB No.: NCR246033).

### 2.4. Data Management and Analysis

#### 2.4.1. Student Demographics & Food Security

Student demographic information was collected through the survey administered in Fall 2024. We measured household food security status using the United States Department of Agriculture Six-Item Short Form of the Food Security Survey Module, asking students to reflect on the previous 30 days at each point of data collection [39]. Students received a raw score of zero to six points. Those scoring zero or one point at all time points of data collection were classified as persistently experiencing food security throughout the Fall 2024 semester [39]. Those scoring two to six points at any point of data collection were classified as experiencing food insecurity at any time point in the Fall 2024 semester [39].

We also collected information regarding year of enrollment in undergraduate studies, housing status (on-campus or off-campus), meal plan enrollment (including access to dining halls through “meal swipes” and “dining dollars” to spend at on- and off-campus partner food retail locations), gender identity, racial and ethnic identity, international student status, parent educational attainment, financial aid receipt, dietary restrictions, employment status, receipt of the SNAP in childhood/adolescence (prior to university enrollment), current SNAP enrollment, and use of charitable food supports over the previous 30 days. We summarized these characteristics using descriptive statistics in Stata MP (Version 18.0, StataCorp, College Station, TX, USA).

#### 2.4.2. Transcription and Coding

All audio recordings were transcribed by a professional transcription company and checked for accuracy by the research assistants. Close readings for all transcripts were completed by each research assistant and the principal investigator, followed by each team member completing line-by-line inductive coding to develop a preliminary list of codes. The research team then met to discuss preliminary codes and code meanings, resulting in a preliminary collaborative list of n = 116 codes. Then, codes were collapsed, refined, and mapped across five code groups resulting in n = 72 total codes. Code groups included: (1) student characteristics & living situation (n = 7 codes); (2) food access & acquisition (n = 30 codes); (3) eating behaviors & preferences (n = 14 codes); (4) food experiences & reflections (n = 10 codes); and (5) barriers & stressors (n = 11 codes).

Each research assistant piloted the codebook on two transcripts. The research team then met to discuss discrepancies in code use and to refine code definitions. An additional two transcripts were then coded using the refined code definitions, and the research team met to discuss and compare codes. Agreement was achieved with code use, resulting in one lead research assistant coding all transcripts (n = 22), and two additional researcher assistants coding n = 11 transcripts each. This resulted in every transcript being double-coded, with code differences being resolved through comparison and discussion in weekly team meetings. Coding was completed over a 5-week period, and all coding was completed in ATLAS.ti (Version 25.0.1, ATLAS.ti, Scientific Software Development GmbH, Berlin, Germany, 2025).

#### 2.4.3. Thematic Analysis

The research team completed a thematic analysis using a phronetic iterative approach. Following inductive coding, preliminary inductive themes identified through cross-code inquiries, memos and team discussions were deductively organized across the socio-ecological conceptual model of determinants to college student food access developed by Landry and colleagues [4]. Specifically, the research team mapped all identified themes across four total domains informed by Landry et al.’s conceptual model. For the purposes of our analysis, we collapsed the community and institutional determinants of Landry et al.’s framework into a single domain. Additionally, a comparative thematic analysis was completed comparing the experiences of undergraduates reporting food security vs. food insecurity.

### 2.5. Reflexivity in the Research Process

To promote rigor throughout the research process, the project was co-developed and completed with a research team comprising undergraduate and graduate student research assistants with varying degrees of experience either supporting student food access on campus or bringing lived experience of food insecurity to their involvement in the research project. In addition, the student basic needs program associate, first generation program associate, and dining director at the university were important stakeholders and research team members who informed data collection tools and interpretation of themes and recommendations given their day-to-day expertise and experiences helping students achieve food security on campus. The principal investigator is a university faculty member who currently experiences food security. During her undergraduate and graduate studies, she experienced marginal food security. Given her positionality to the work, the principal investigator did not conduct any interviews or have any interactions with study participants. Throughout interviews and data analysis, research members engaged in reflective memoing to bracket and reflect on assumptions about how their own lived experiences related to the data collection process and emerging themes. Throughout the research process, weekly meetings allowed for the research assistants and the principal investigator to discuss reflections and minimize bias in the research process.

## 3. Results

### 3.1. Student Characteristics

We interviewed n = 10 undergraduate students experiencing food security and n = 12 undergraduates experiencing food insecurity (Table 2). Students identified across a diverse mix of races; 32% (n = 7) as white, 18% (n = 4) as Black or African American, 18% (n = 4) as Asian or Asian American, 14% (n = 3) as another race, and 18% (n = 4) as two or more races. The majority identified as non-international status (82%, n = 18) and female (68%, n = 15). Most students interviewed were enrolled in their first year (41%, n = 9), including n = 8 students (67%) who reported experiencing food insecurity. Nearly all students lived in campus housing (86%, n = 19) and were enrolled in an unlimited meal plan (45%, n = 10) meaning they could eat in a campus dining hall once per hour every day. A total of n = 14 students (64%) received financial aid, including n = 8 students (36%) who received Pell Grants. Only n = 2 students (9%) reported receiving SNAP benefits at the time of the interview, but n = 5 (23%) students reported growing up in a household that received SNAP. No students in the sample reported using charitable food supports on- or off-campus within the prior 30 days.

### 3.2. Thematic Findings

We identified nine themes across four domains describing the determinants of food insecurity, including: (1) individual determinants (n = 3 themes); (2) interpersonal determinants (n = 2 themes); (3) institutional and community determinants (n = 3 themes); (4) policy determinants (n = 1 theme; Table 3). Across domains and themes, differences among students experiencing food insecurity compared to food security emerged and are illustrated throughout the narrative review of thematic findings.

### 3.3. Domain 1: Individual Determinants of Food Security

#### 3.3.1. Domain 1, Theme 1: Personal Resources for Cooking and Groceries Are Not Available to Those with Unmet Food Needs

All students living on campus described barriers to accessing a kitchen and cooking equipment, but this challenge was more complex among students experiencing food insecurity. For example, students experiencing food insecurity commonly described lacking financial resources outside of their meal plan, preventing them from buying ingredients to cook simple meals (Table 4, quote 1). Additionally, communal kitchens across campus housing were described as inconsistently stocked, requiring students to use their own cooking equipment.

To gain financial resources for food, some students experiencing food insecurity described picking up additional work hours during the semester and academic breaks (i.e., winter break). Further, one student illustrated unintended health consequences of food insecurity, including loss in appetite and micronutrient deficiencies (Table 4, quote 2). In contrast, students experiencing food security commonly had multiple financial resources, including scholarships, personal savings, family support, and part-time employment, describing their earnings as supplemental income for both essential and non-essential needs (Table 4, quote 3).

#### 3.3.2. Domain 1, Theme 2: Time Scarcity Requires Convenience to Be Prioritized

All students in our sample described time scarcity as a challenge throughout the semester. During times of higher academic demands, such as midterms and finals, students illustrated tradeoffs made between being able to eat nourishing meals and prioritizing studying. Students experiencing food security more commonly described successful time management strategies, such as buying quick snacks to consume between different obligations or prioritizing time to prepare meals during busier weeks (Table 4, quote 4). No students experiencing food insecurity described reliable coping and management strategies when faced with time scarcity and commonly described skipping meals when their schedule (i.e., academic and work) became too demanding.

#### 3.3.3. Domain 1, Theme 3: Unmet Food and Dietary Needs Compromise Students’ Physical and Mental Health, Negatively Affecting Academic Performance

Most students, regardless of food security status, reported that university meal plans often failed to meet their food preferences and dietary needs. At the university, there are different meal plans available to students that offer a combination of “meal swipes” to access an all-you-can-eat dining hall and “dining dollars” which are equivalent to USD to be spent at on- and off-campus dining. Meal plans were priced between $2200–$2940/semester in Fall 2024, and students living on-campus were required to pay for a meal plan. First-year students were required to enroll in the “All Access” meal plan, which provides unlimited meal swipes for dining halls and $240–$340 in dining dollars per semester.

However, students experiencing food insecurity shared that they could always eat on the “All Access” plan, but they did not find the food available in dining halls to meet their health needs (Table 4, quote 5). Other students described physical health challenges from consuming the food available in the dining halls and on-campus food retailers, which at times also resulted in mental health consequences (Table 4, quote 6). Additionally, students experiencing food insecurity without unlimited meal swipes noted stress and mental health challenges from not knowing where their next meal would come from, which distracted them from fully focusing on their academic demands (Table 4, quote 7).

### 3.4. Domain 2: Interpersonal Determinants of Food Security

#### 3.4.1. Domain 2, Theme 4: Family Financial Support Promotes Food Security but Is Not Accessible to All

Reliable and consistent financial support from family members was available for nearly all students experiencing food security, while all students experiencing food insecurity described little to no financial support from family members. For example, one student experiencing food security described having access to their parents’ financial resources to be able to supplement their food budget however needed, without even asking (Table 5, quote 1). Ease of access to money from family emerged as one of the strongest protections against food insecurity. On the contrary, students experiencing food insecurity described shame and guilt asking for additional financial support from family, especially when also enrolled and paying for a meal plan (Table 5, quote 2).

#### 3.4.2. Domain 2, Theme 5: Informal Support from Friends and Coaches Can Help Fill Institutional Gaps

Among students experiencing food insecurity, it was rare to describe food support coming from friends, but a few students noted occasionally receiving snacks from their friends in their social network (Table 5, quote 3). Students experiencing food security commonly described sharing meals and cooking with friends both on campus and off campus (Table 5, quote 4). Among the few student athletes in our sample (n = 4), all described how their university coaches would regularly help them meet their food needs by providing snacks, or even meals. One student athlete emphasized the importance of this supplement when campus dining options regularly closed prior to the end of practice (Table 5, quote 5).

### 3.5. Domain 3: Institutional and Community Determinants of Food Security

#### 3.5.1. Domain 3, Theme 6: Campus Dining Operations Are Not Always Aligned with Student Realities and Preferences but Do Help the Most Vulnerable Meet Their Needs

All students described the operating hours of dining halls as misaligned with their schedules. Among students experiencing food insecurity, the challenges were more severe as work and class schedules both created conflicts (Table 6, quote 1), while students experiencing food security more commonly cited challenges only with class schedules, especially during midterms and finals (Table 6, quote 2). Similarly to operating hours, all students, regardless of food security status, preferred to have more dining dollars than meal swipes due to its flexibility for food acquisition on- and off-campus (Table 6, quote 3). However, students experiencing food insecurity noted that unlimited meal swipes provided stable and consistent access to food, even when the options were not always preferred. One student specifically noted how this consistency was not present in their household growing up due to their limited food budget (Table 6, quote 4). Yet, this student still reported experiencing food insecurity at the university, suggesting that dining hall options may not adequately meet their personal food needs.

#### 3.5.2. Domain 3, Theme 7: High Costs of Living and Transportation Barriers Hinder Food Access off Campus

Many students described how the high cost of living in an urban setting (e.g., Washington, DC) motivated them to take advantage of food resources provided by the university. Students experiencing food insecurity frequently compared themselves to their peers with higher financial security and perceived them as easily able to navigate high costs of living through resources such as credit cards or family support (Table 6, quote 5). Rather than reporting feelings of stigma, students emphasized the need to make the most of campus dining resources in place of off-campus options.

Additionally, all students, regardless of food security status, illustrated challenges in reaching the only grocery store that accepted dining dollars due to the reliance on public transportation for accessibility (Table 6, quote 6). Many students said they would prefer a grocery store partnership closer to campus so it would be easier to use meal plan resources without needing to travel far. Some upperclassmen students in the sample recalled when a nearby grocery store accepted dining dollars and how this proximity more easily supported their food needs.

#### 3.5.3. Domain 3, Theme 8: Students Make Limited Use of Formal Food Assistance Resources and Instead Turn to Campus Events as Their Most Familiar and Accessible Source of Free Food

On-campus, there is a food pantry available to all full- and part-time students, yet no students in our sample reported using the food pantry within the last 30 days. Only one student in the sample (they experienced food insecurity) described knowing about the food pantry and ever using it. Similarly, no students interviewed were aware of a program on campus collecting and redistributing extra meal swipes, and no students reported awareness or use of off-campus free food resources, such as food pantries in the community. Instead, students, particularly students experiencing food insecurity, noted many campus events available to students provided free food, which was a source of nourishment for them (Table 6, quote 7).

### 3.6. Domain 4: Policy Determinants of Food Security

Domain 4, Theme 9: The Supplemental Nutrition Assistance Program (SNAP) Is Underutilized on Campus

Only n = 2 students in our sample, both reporting food insecurity, stated receiving and using SNAP at the time of this study. Some students experiencing food insecurity who reported that their family received SNAP benefits did not think they would qualify because their family still received SNAP benefits (Table 6, quote 8). Across the sample, few students were aware of SNAP as a federal supplemental food resource, and most students did not know where and how to receive additional information.

## 4. Discussion

This qualitative study offers insight into how undergraduate students at a private, urban U.S. university experience food insecurity and navigate interpersonal, institutional, community, and policy-level determinants within the context of their daily lives. Findings illustrate that food insecurity among college students is multidimensional and shaped by factors beyond the mere presence of food on campus. Across four thematic domains, students described barriers affecting access, availability, stability, utilization, and agency. At the individual level, limited financial resources and time scarcity constrained food access and utilization. At the interpersonal level, lack of family financial support reduced stability and agency, leaving students unable to secure food beyond what was provided through meal plans. At the institutional and community level, dining hall hours and food options compromised food utilization and agency, while transportation barriers the area grocery store limited availability for students seeking to use dining dollars on groceries. Awareness gaps further prevented some students from accessing campus-based free food resources. Finally, at the policy level, restrictive SNAP eligibility and limited program knowledge hindered access to supplemental benefits among students who may have qualified. Taken together, these findings demonstrate that food insecurity among college students reflects complex intersections of financial constraints, institutional structures, and policy barriers, causing certain dimensions of food security to be particularly vulnerable.

Students in our study consistently identified financial strain as a primary individual and interpersonal determinant of food insecurity, even among those enrolled in a meal plan. Rising tuition, housing costs, and mandatory meal plan fees reduced disposable income for groceries and other essentials, limiting economic access to food beyond what was included in the cost of attendance. This finding aligns with prior quantitative and qualitative research showing that insufficient financial resources are a key driver of food insecurity among college students [4,9,12,22]. Notably, U.S. studies indicate that students receiving financial aid, including Pell Grants, are at higher risk of food insecurity compared to peers with greater financial security, suggesting that current aid packages may be inadequate to meet basic needs such as food [5,8,12,40,41]. These realities also highlight that meal plans alone cannot fully ensure food security, which is similar to research from Mei and colleagues that found 14% of undergraduates (n = 1033) enrolled in an unlimited meal plan reported experiencing food insecurity [23]. Together, these findings underscore that financial access, while necessary, is insufficient to secure all dimensions of food security, particularly when students lack agency or the ability to obtain foods that meet their health, cultural, or dietary needs.

One reason meal plans, including unlimited options, may not lead to universal food security is that they primarily guarantee financial access to food on campus but cannot guarantee dining hours that align with students’ schedules, nor do they always provide sufficient variety to meet cultural, dietary, and personal preferences [23]. In our study, students experiencing both food security and insecurity reported challenges with dining hall hours, but only those with additional financial resources could navigate alternative options off-campus. Notably, most students in our study who reported food insecurity (67%) were first-year students enrolled in unlimited meal plans. A cross-sectional analysis of first-year college students in the U.S. found that food insecurity was associated with students using fewer meal swipes, and it was working students who used their meal plan less [42]. Extending dining hours or introducing alternative options for using meal swipes (e.g., grab-and-go models) may help reduce unmet food needs among meal plan users at risk for food insecurity. However, such modifications address only the availability and access dimensions of food security; they do not resolve limitations related to agency and utilization, including nutritional quality and cultural appropriateness. Further research is needed to determine whether improving meal-plan flexibility can meaningfully enhance students’ agency and utilization within campus dining systems.

In our sample, some students experiencing food insecurity acknowledged that food was consistently accessible and available to them, but they described not always feeling they had meaningful choice or variety to meet their needs. This reflects limited agency and utilization, key dimensions of food security, which may persist even when access and availability to food is assured. Evidence indicates that students value health, variety, and choice in dining options, and perceive reductions in these elements as barriers to satisfaction [43]. In our study, students also described barriers to utilizing available food in ways that supported health and well-being. Students experiencing both food security and insecurity expressed concerns about the nutritional quality and appeal of dining hall options. However, students with financial flexibility could overcome these limitations by purchasing food off-campus, whereas those experiencing food insecurity were unable to do so. These patterns illustrate that when students are unable to utilize campus dining systems to meet dietary and cultural food needs, food security can be undermined even when food access is “unlimited”. This gap underscores the importance of designing campus dining systems that not only provide consistent access but also offer foods that align with health, dietary, and cultural preferences. This finding is consistent with a cross-sectional survey of n = 409 college students in Australia where they reported healthfulness and quality of food as their top-rated preferences [44]. Without addressing these utilization and agency-related barriers, unlimited meal plans will be unable to guarantee food security across all dimensions.

Our research underscores the importance of capturing the multidimensional nature of food insecurity among college students. However, existing USDA-validated tools, such as the 18-item Household Food Security Survey Module (10-item version for individuals without children) and the Six-item Short Form, primarily assess economic access to food [39]. These measures do not fully account for other critical dimensions of college food security, including agency, utilization, stability and availability [45]. Moreover, recent studies using these validated tools alongside cognitive interviews reveal that college students often misinterpret survey questions, resulting in inaccurate prevalence estimates and questioning the validity of these measures in higher education contexts [46,47]. As potential new measurements are developed and validated, they should prioritize the inclusion of additional dimensions of food insecurity (i.e., utilization, agency, stability, and availability) to best capture the realities of college students.

This study has limitations that should be considered alongside its strengths. First, study participation was voluntary, which may have introduced potential selection bias. Interview responses may also have been influenced by social desirability bias as participants may have underreported stigmatizing experiences despite emphasis on confidentiality. Second, data collection occurred during the Fall semester and extended into the winter break which may have constrained participation and influenced students’ experiences due to academic pressures or environmental changes. Third, most interview participants were first-year students (41%, n = 9), including n = 8 students (67%) who reported food insecurity, which may not reflect the experiences of upperclassmen. Fourth, we were unable to analyze thematic differences between the experiences of persistent and episodic food insecurity, which would have provided deeper insight into the stability dimension of food security. Finally, the sample was drawn from a single urban, private institution, limiting generalizability to campuses with different contexts. Findings may be most transferable to students attending similar private institutions with comparable demographics and food environments. However, these limitations were offset by several strengths. We were able to compare themes by food security status (food secure vs. food insecure), gain insight into why students with unlimited dining plans experienced food insecurity (partly due to our larger first-year sample) and include perspectives from financial aid recipients and employed students, both factors known to increase risk of food insecurity.

Future research should extend to other universities to better understand how differences in institutional context shape student food insecurity (e.g., private vs. public universities). Additionally, future studies should prioritize larger and more diverse samples to examine potential differences across student subgroups (e.g., international students vs. U.S. students). Findings from this study also highlight the need for institutional strategies that move beyond food access alone; future changes should be paired with evaluations to help identify which changes and investments best support students across the multiple dimensions of food security. For example, universities could adopt greater variety and cultural relevance within meal plans, modify dining hall hours to better align with student schedules, and create improved mechanisms for using campus dining dollars at off-campus grocery stores or culturally diverse food outlets. Improvements to transportation access, such as shuttle routes to grocery stores, may further support students’ ability to obtain needed foods. In addition, universities could strengthen communication and outreach about campus food pantries, emergency meal programs, community-based food resources, and offer assistance with SNAP screening and application through student support offices. Addressing these institutional and policy areas are essential to supporting all dimensions of food security among college students.

## 5. Conclusions

This study provides insight into the multidimensional nature of food insecurity among college students and the intersecting individual, interpersonal, institutional, community, and policy factors that shape it. Findings highlight that meal plans alone may not ensure food security when barriers related to food agency, utilization, and availability persist. Addressing these gaps will require campus strategies that go beyond access to include flexibility, variety, and alignment with students’ schedules and food preferences. Future research should examine interventions that integrate all six dimensions of food security and inform institutional policies that promote health, equity, and academic success.

## Figures and Tables

**Table 1:** Example In-Depth Interview Questions and Probes by Question Domain.

Question Domain	Example Questions	Example Probes
Food Acquisition Habits	Take a minute a think about all the places in a week you typically go to get food. Now, can you please walk me through all the different places you go to get food?	Can you please describe to me the types of foods you get at each of those places?
	What factors do you consider when deciding which place you will go to get food?	Of the factors you named, such as [insert factors named], which are the most important to you? Why?
	Can you please walk me through and describe to me who helps you get the food that you need throughout the semester?	Does anyone give you money for food? Do you fully support yourself?
Food Management Strategies	Can you tell me about some of the things that make it hard for you to get all the food you want and need during the semester?	Over a semester, if your resources for food become tight, how do you manage this?
	Can you please tell me what you know about programs at GW that help students meet their food needs, other than meal plans?	What other resources, if any, do you wish were available to students to better meet the food needs of students?
	Can you please tell me what you know about programs outside of the GW community that can help students meet their food needs?	How, if at all, do these programs help you meet your food needs?
Food Access, Health, Academics, and Social Life	How would you describe your relationship with food?	How, if at all, has this relationship changed since starting undergrad at GW?
	Can you describe to me your typical routine at a mealtime?	How does mealtime make you feel? What do you enjoy about it? What is challenging?
	How does your relationship with food impact your overall academic performance?	How, if at all, do your school demands impact what you eat? How does this make you feel?

**Table 2:** Characteristics of Undergraduate Students Completing In-Depth Interviews (n = 22).

Characteristic	Overall (n = 22, 100%)		Students Experiencing Food Security (n = 10, 45%)		Students Experiencing Food Insecurity (n = 12, 55%)	
Demographic characteristics						
Race/ethnicity, n (%)						
White	7	32%	4	40%	3	25%
Black or African American	4	18%	2	20%	2	17%
Asian or Asian American	4	18%	3	30%	1	8%
Other Race	3	14%	0	0%	3	25%
Two or more races	4	18%	1	10%	3	25%
International student status, n (%)						
Yes	3	14%	2	20%	1	8%
No	18	82%	8	80%	10	83%
Refuse to answer	1	5%	0	0%	1	8%
Gender identity, n (%)						
Woman	15	68%	8	80%	7	58%
Man	5	23%	2	20%	3	25%
Other	2	9%	0	0%	2	17%
Mean age, y (SD)	18.7	(0.98)	19.3	(0.98)	18.3	(0.87)
Campus life characteristics						
Enrollment year, n (%)						
1st year	9	41%	1	10%	8	67%
2nd year	7	32%	5	50%	2	17%
3rd year	5	23%	3	30%	2	17%
4th year	1	5%	1	10%	0	0%
Housing, n (%)						
On-campus housing	19	86%	8	80%	11	92%
Off-campus housing	3	14%	2	20%	1	8%
Student athlete, n (%)						
Yes	4	18%	3	30%	1	8%
No	18	82%	7	70%	11	92%
Meal plan and diet characteristics						
Meal plan enrollment, ^a^ n (%)						
All Access	10	45%	2	20%	8	67%
Revolutionary 85	7	32%	5	50%	2	17%
Block 90	2	9%	1	10%	1	8%
Unsure	1	5%	1	10%	0	0%
Not enrolled	2	9%	1	10%	1	8%
Dietary Restrictions, n (%)						
No restrictions	18	82%	9	90%	9	75%
At least 1 restriction	4	18%	1	10%	3	25%
Socioeconomic characteristics						
Any Financial Aid Receipt, ^b^ n (%)	14	64%	5	50%	9	75%
Pell Grant Receipt, ^c^ n (%)	8	36%	1	10%	7	58%
Employment Status, n (%)						
Unemployed	9	41%	4	40%	5	42%
Employed part time	13	59%	6	60%	7	58%
Parent educational attainment, n (%)						
High school or less	4	18%	1	10%	3	25%
Some college or Associate	1	5%	1	10%	0	0%
Bachelor’s degree	5	23%	2	20%	3	25%
Graduate degree	11	50%	6	60%	5	42%
Unsure	1	5%	0	0%	1	8%
SNAP Receipt ^d^, n (%)						
Prior to university	5	23%	1	10%	4	33%
Current	2	9%	0	0%	2	17%
Use of charitable food support in the last 30 days, n (%)						
On-campus food pantry	0	0%	0	0%	0	0%
Off-campus food pantry	0	0%	0	0%	0	0%
Donated meal swipes ^e^	0	0%	0	0%	0	0%

^a^ Students living on campus are required to select a meal plan. “All Access” provides unlimited meal swipes and $240–$340 in dining dollars per semester. All 1st year undergraduates must enroll in the “All Access” plan. “Revolutionary 85” provides 85 days of unlimited meal swipes per semester and $840 dining dollars per semester. “Block 90” provides approximately 5 meal swipes per week and $1030 in dining dollars per semester. ^b^ Any financial aid includes federal student loans that will be repaid by the student following degree completion and scholarships which are not repaid. ^c^ The Pell Grant is a needs-based scholarship for low-income students identified as having exceptional financial need. ^d^ The Supplemental Nutrition Assistance Program (SNAP) is supplemental financial support provided by the federal government in the U.S. to buy groceries at authorized retailers. Individuals and households must meet income and eligibility criteria to participate. ^e^ Students enrolled in a Block meal plan (i.e., “Block 90”) can donate extra swipes at the end of the semester to redistribute to other students in-need of food assistance.

**Table 3:** Domains and Themes Describing the Individual, Interpersonal, Institutional and Community, and Policy Determinants of Food Security among Undergraduates at a Private, Urban University.

Domain	Description of Domain	Themes
Individual determinants	Students’ food and nutrition needs, and the extent to which they can meet those needs given their personal resources, time constraints, and daily schedules.	Personal resources for cooking and groceries are not available to those with unmet food needs.
		2. Time scarcity requires convenience to be prioritized.
		3. Unmet food and dietary needs compromise students’ physical and mental health, negatively affecting academic performance.
2. Interpersonal determinants.	Students’ access to emotional, social, and material support for meeting their food needs through relationships with family, friends, mentors, coaches, and peers.	4. Family financial support promotes food security but is not accessible to all.
		5. Informal support from friends and coaches can help fill institutional gaps.
3. Institutional and community determinants	Students’ ability to access campus and community food environments, including the alignment of dining hours with their schedules, the availability and quality of on-campus food options, and the accessibility of off-campus food retail. Students’ awareness and use of free-food and charitable support resources.	6. Campus dining operations are not always aligned with student realities and preferences, but do help the most vulnerable meet their needs.
		7. High costs of living and transportation barriers hinder food access off campus.
		8. Students make limited use of formal food assistance resources and instead turn to campus events as their most familiar and accessible source of free food.
4. Policy determinants	Students’ understanding of federal nutrition assistance programs and their ability to meet food needs within the financial constraints and policy structures of a high-cost higher education environment.	9. The Supplemental Nutrition Assistance Program (SNAP) is underutilized on campus.

**Table 4:** Illustrative Quotes: Individual Determinants of Food Security.

Quote Number	Quotes
1	“No [I don’t cook], because I need to get groceries and I don’t have the money to buy groceries. I know in the past GW used to partner with Whole Foods to be able to use our dining dollars there, but I don’t think we have that this year. So, if you go to Whole Foods, you have to spend your own money, which I don’t have a lot of. So, I’ve never been able to like, really buy like, you know, eggs so I can make breakfast or like one of those, like, instant meals that you heat up for like five minutes on the stove. And then also, even though we have a kitchen there aren’t like communal pots and pans. So, you have to like get your own” [ID#82, undergraduate experiencing food insecurity]
2	“But like in the beginning of semester, it was really bad. Like, I was thinking about food all the time, I didn’t have enough I guess. So, I would like constantly think how hungry I am or I’m not, or like what I’m going to eat, how much…as the semester continued I worked more, and I worked during the breaks. So, I now could satisfy, like kind of my needs. But if being honest, I need less. I don’t know if my stomach shrank or something. I’m not that hungry anymore. Like at some point, I just lost all of my appetite…I’m also iron deficient and vitamin D deficient. But I brought those vitamins from home. But as soon as they finished, I didn’t buy new ones” [ID#75, undergraduate experiencing food insecurity]
3	“I do have a little bit of money coming in. Plus, I’m on scholarship here and they did just award me with kind of like a stipend. So, it’s like a $1500 a semester thing. That’s really been helpful. I haven’t had to stress out about not being able to buy things for myself. So, it’s a lot of things. I would say scholarships, the dining plan money, my own savings, money coming in from my job.” [ID#31, undergraduate experiencing food security]
4	“So sometimes that’s challenging where it’s like, okay, like, what’s the quickest thing I can get? Or do I have to like, prepare something ahead of time so I know I’m going to be able to eat at least something. So, it’s about time management” [ID#30, undergraduate experiencing food security]
5	“I mean, I can eat because I can swipe once an hour. I’m completely able to get meals…but it’s not always the meals that I want or need to have health wise[…]I feel like I could be eating healthier. There are better options outside of GW dining. Like better than the options we currently have.” [ID#33, undergraduate experiencing food insecurity]
6	“I just always have a ton of stomach problems coming out of [the dining hall]. Like, within a half an hour my stomach is hurting[…]especially last year, like every day, feeling like that really started to weigh me down. I experienced a lot of bloating. And I think for me that hit hard mentally because although I wasn’t like gaining weight, just like in my mind, I didn’t look great and that wasn’t fun. And for me, it just felt like there wasn’t really a way around that” [ID#44, undergraduate experiencing food insecurity]
7	“I think when you’re so stressed about knowing where your next meal is coming from, you don’t know where you’re going to get your next meal. You don’t know if you have the funds for food…that just always takes up more space than classes and what you should be focused on. Just because it’s your physical health that has to come first” [ID#440, undergraduate experiencing food insecurity]

**Table 5:** Illustrative Quotes: Interpersonal Determinants of Food Security.

Quote Number	Quotes
1	“So, I have my own banking, checking and savings account. So, some of [my money for food] is coming from there. Some of it’s coming from just cash that I have. I do have access in some ways to my parents’ accounts, so I can use that as well” [ID#43, undergraduate experiencing food security]
2	“[My parents] had agreed to just send money over when I get groceries, like once a month. But that hasn’t always happened. And I feel like bad for asking. Because I don’t want to. I’m trying not to spend a ton, and they’ll talk to me about spending my own money. Especially on food, because I have the meal plan” [ID#33, undergraduate experiencing food insecurity]
3	“‪I start foraging from like other friends sometimes, or saving snacks from dining halls, like an apple or a banana. And then if I just need something then I would eat that” [ID#484, undergraduate experiencing food insecurity]
4	“I cook with my roommates. We all brought a lot of kitchen supplies and stuff to school. And my two of my roommates are also on the team with me, so…we’re kind of on the same schedule. So, a lot of times we’ll cook with each other” [ID#301, undergraduate experiencing food security]
5	“Yeah, we had Sunday practice. We would get back to campus at like 8:50 pm. The dining hall closed at 9:00 pm and we would walk in there we’d be like please, do you have any food for us and they’re like already closing at like 8:45 pm. Like they’re trying to get people out and like kicking them out. So, it got to the point where we would finish practice and then [my coach] would drive us over to Tropical Smoothie and, like, pay for a meal for all of us. But that was every single Sunday. Like we have no options for anywhere to get dinner after practice. Crazy. How is it you can’t, like, supply food for your athletes?” [ID#31, undergraduate experiencing food security]

**Table 6:** Illustrative Quotes: Institutional, Community, and Policy Determinants of Food Security.

Quote Number	Quotes
1	“So, I’ll have to, you know, head down to the dining hall. I think the dining hall reopens at like 4:30 pm, but I got to be at work at 5:00 pm, and then by the time I’m off work, the dining hall is closed. So, it’s like I have that very tight window to get my dinner, and if I miss it, then it’s like back to the snacks” [ID#47, undergraduate experiencing food insecurity]
2	“But like when the dining halls would close around finals sometimes it was difficult because I might go to a dining hall and there wasn’t food that I liked, and there were maybe only 1 or 2 options to choose from. So, this year, like during finals, I haven’t gone to the dining halls as much just because I knew that there wouldn’t be as much open. So, I guess that put a little bit more pressure on me to eat out or make things on my own” [ID#52, undergraduate experiencing food security]
3	“I still don’t need all those swipes. I wish they would let me choose the money, let me grocery shop, let me like, figure out my own meals. Because just giving us all those swipes, or at least for me, giving me all the days to go there, it’s not helpful. And like, I don’t want a cheeseburger, I don’t want Halal Shack. I want to get my own food” [ID#31, undergraduate experiencing food security]
4	“I would honestly say I eat more at GW than I did at my house just because I have those, you know, unlimited swipes. I can get as much as I need. Whereas at home, you know, we have a fixed budget on the amount of food. There’s only so much you can eat until you know the next check comes in” [ID#470, undergraduate experiencing food insecurity]
5	“With my family, because there is not as much as some other people have and, you know, I notice this. I have friends who can just charge a credit card or just have higher means than me. I can’t do that, and I just have to deal with it. I have to be aware of the price for things versus other people. I’m not going to say everybody is not aware of the price, but there’s definitely a difference” [ID#24, undergraduate student experiencing food insecurity]
6	“I do my grocery shopping on Fridays…because I don’t have any classes on Fridays. However, taking the bus all the way to the Georgetown Safeway. First of all, you have to wait for the bus. Then you get there, you have to buy everything, and then you have to wait for the bus and then come back. That can suck time…the distance and the time to get to a place that actually takes your dining dollars can make it really difficult” [ID#436, undergraduate experiencing food security]
7	“Like this past maybe ten days I couldn’t cook anything. But again, like thankfully there were so many like end of semester events that I’ve been just eating all around campus” [ID#75, undergraduate experiencing food insecurity]
8	“I know SNAP does help and my family relies on it, but it’s connected with my family, so I personally can’t use it like while I’m at school. So, it’s like a different situation” [ID#484, undergraduate experiencing food insecurity]

## Data Availability

The raw data supporting the conclusions of this article will be made available by the authors on request due to ethical and confidentiality considerations associated with in-depth qualitative interviews.

## Abbreviations

The following abbreviations are used in this manuscript:

GW	George Washington University
SNAP	Supplemental Nutrition Assistance Program
U.S.	United States of America
USDA	United States Department of Agriculture
